# Bioinformatics analysis of transcriptional regulation of circadian genes in rat liver

**DOI:** 10.1186/1471-2105-15-83

**Published:** 2014-03-25

**Authors:** Tung T Nguyen, John SA Mattick, Qian Yang, Mehmet A Orman, Marianthi G Ierapetritou, Francois Berthiaume, Ioannis P Androulakis

**Affiliations:** 1 Chemical and Biochemical Engineering Department, Rutgers the State University of New Jersey, Piscataway, NJ 08854, USA; 2 BioMaPS Institute for Quantitative Biology, Rutgers the State University of New Jersey, Piscataway, NJ 08854, USA; 3 Biomedical Engineering Department, Rutgers the State University of New Jersey, Piscataway, NJ 08854, USA

**Keywords:** Circadian rhythm, Microarray analysis, Gene expression, Consensus clustering, Promoter analysis, Transcription factor, Circadian regulation

## Abstract

**Background:**

The circadian clock is a critical regulator of biological functions controlling behavioral, physiological and biochemical processes. Because the liver is the primary regulator of metabolites within the mammalian body and the disruption of circadian rhythms in liver is associated with severe illness, circadian regulators would play a strong role in maintaining liver function. However, the regulatory structure that governs circadian dynamics within the liver at a transcriptional level remains unknown. To explore this aspect, we analyzed hepatic transcriptional dynamics in Sprague-Dawley rats over a period of 24 hours to assess the genome-wide responses.

**Results:**

Using an unsupervised consensus clustering method, we identified four major gene expression clusters, corresponding to central carbon and nitrogen metabolism, membrane integrity, immune function, and DNA repair, all of which have dynamics which suggest regulation in a circadian manner. With the assumption that transcription factors (TFs) that are differentially expressed and contain CLOCK:BMAL1 binding sites on their proximal promoters are likely to be clock-controlled TFs, we were able to use promoter analysis to putatively identify additional clock-controlled TFs besides PARF and RORA families. These TFs are both functionally and temporally related to the clusters they regulate. Furthermore, we also identified significant sets of clock TFs that are potentially transcriptional regulators of gene clusters.

**Conclusions:**

All together, we were able to propose a regulatory structure for circadian regulation which represents alternative paths for circadian control of different functions within the liver. Our prediction has been affirmed by functional and temporal analyses which are able to extend for similar studies.

## Background

The circadian clock is one of the most critical biological regulators for all living entities controlling behavioral, physiological and biochemical processes [1]. Acting like a multifunctional timer with a roughly 24-h cycle [2], the circadian clock offers fitness advantages by endowing organisms with anticipatory mechanisms predicting periodic events, as opposed to responding in a reactive way to external signals [3]. Robust circadian regulation is associated with fitness, while abnormal rhythms which are characteristic of stress are often linked with illness [4,5]. As an example, metastatic colorectal cancer patients with severe alterations of the rest-activity circadian rhythm were predicted to have a fivefold increase in the risk of death compared to a normal rest-activity pattern [6].

In mammals, the circadian clock is composed of auto-regulated transcription-translation feedback loops whose primary elements are transcription factors (TFs) including the basic helix-loop-helix transcription factor CLOCK and the aryl hydrocarbon receptor nuclear translocator-like BMAL1, which drive the expression of period (PER) and cryptochrome (CRY). PER and CRY form the negative limb of the feedback loop, repressing the expression of CLOCK–and BMAL1–induced transcription [7]. Evidence suggests that circadian gene expression is achieved through a transcriptional cascade in which the expression of other TFs responsible for the majority of rhythmic tissue-specific expression are primarily driven directly by the main clock transcription factor complex CLOCK:BMAL1 [8]. Recently, this hypothesis has been reinforced by experiments showing that TFs whose expression patterns exhibit daily oscillation are direct targets of the CLOCK:BMAL1 complex [9,10]. However, those studies only explored the transcriptome with one main clock transcription factor mutated and thus the entire picture of how the system behaves or how target genes are controlled is still a question. Consequently, among the aims of our study is to gain insights into the broader transcriptional regulation of circadian rhythms.

Liver is one of the most important organs regulating the metabolic activity of the body as well as producing acute phase proteins (APP, class of proteins responding to inflammatory stressors) in response to external stress [11]. Genome-wide transcriptome-mapping studies have revealed that the fraction of diurnally regulated liver transcripts could amount up to 10%, with genes exhibiting circadian rhythms participate in various functions e.g. cell signaling, energy metabolism, amino acid, lipid and cholesterol metabolism, carbohydrate transport and metabolism, DNA replication, protein synthesis, signal transduction mechanisms, and immune response [8,12-15]. Due to the significance of the circadian periodicity and the severe outcome of disorders following its disruption, a global perspective of the transcriptional dynamics and regulatory structure of circadian genes in liver may offer a molecular framework for studies on the hepatic circadian rhythm gene regulation.

In this study, we address two critical questions including (1) whether we can identify and functionally characterize circadian-regulated genes in an unsupervised manner, and (2) if the potential transcriptional regulatory mechanisms of circadian rhythms can be hypothesized. In order to obtain the necessary samples for analysis, animals were sacrificed at 9 am, 11 am, 1 pm, 5 pm, 1 am, and 9 am (~0, 2, 4, 8, 16, and 24 hr zeitgeber time) via exsanguinations and livers were harvested, allowing for the acquisition of transcriptional data from microarray readouts for those time points. Utilizing our previous consensus clustering [16] and techniques in promoter analysis [17], our results actually demonstrate that clusters of co-expressed genes do vary in a circadian manner and contain functionalities consistent with previous studies including immune function, cell repair, metabolism, and DNA repair. Additional clock-controlled TFs are also predicted. Interestingly, we observed that many clock-controlled TFs relevant to the regulation of genes within a circadian pattern show similar transcriptional dynamics. Furthermore, the predicted regulatory network and regulatory motifs of circadian patterns are shown to be consistent with the biological significance of the corresponding gene set and the circadian time of the transcriptional pattern. Finally, the identified transcription factors are intimately related to innate immunity and response to infection, providing further evidence that circadian rhythms play an important role in recovery from injury. While it is understood that recent evidence points to the important role alternative processes play for driving the induction of oscillations [18-20], the proposed approach presents a generalizable framework for assessing the regulatory structure underlying the transcriptional sources of oscillations.

## Results

### Circadian dynamics and functions

**Figure 1:**
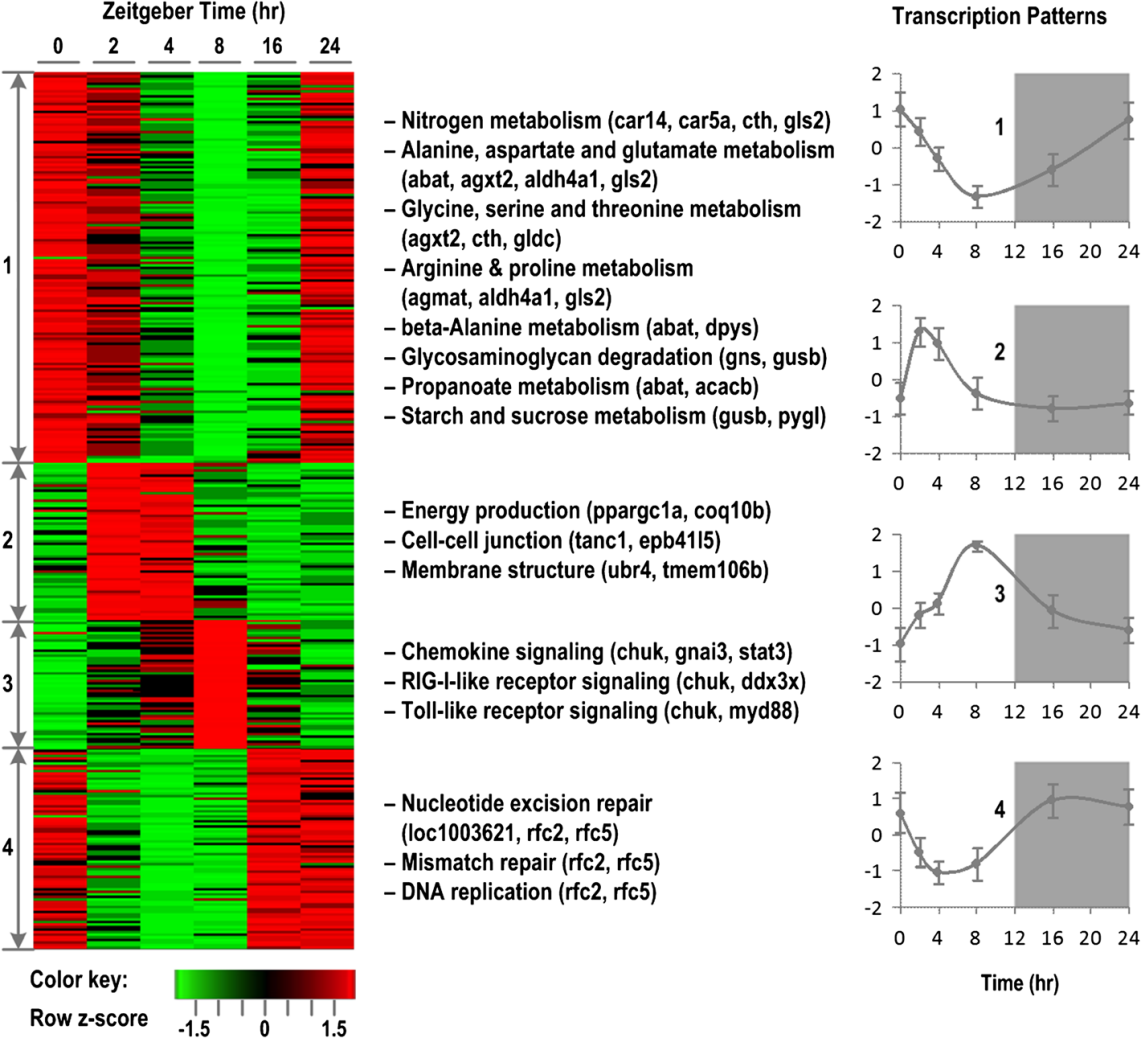
**Circadian patterns in homeostatic rat liver.** Left panel displays the heat map of expression of 153, 64, 52 and 83 probesets in 4 patterns at 0, 2, 4, 8, 16, and 24 hr. Red color indicates the highest level while green indicates the lowest level of expression. Middle panel consists of enriched functions characterizing biological significance of each circadian pattern. Right panel shows the average normalized (z-score) expression profiles of probesets in four corresponding patterns. Error bars are two standard deviations of all probeset transcript levels at each time-point in each corresponding pattern.

Male Sprague–Dawley rats (Charles River Labs, Wilmington, MA) were housed in a temperature-controlled environment (25°C) with a 12-hour light–dark cycle and provided water and standard chow ad libitum. Because the animals are nocturnal, this establishes a 12 hour LD cycle for the animals, where ZT0-ZT12 represents the resting portion of the day, while ZT12-ZT0 represents the active portion of the day. These animals were sacrificed at 9 am, 11 am, 1 pm, 5 pm, 1 am, and 9 am (~0, 2, 4, 8, 16, and 24 hr zeitgeber time) and liver tissues were collected and frozen for microarray analysis (n = 3 per time point per group). The tissues were lysed and homogenized using Trizol, and the RNAs were further purified and treated with DNase using RNeasy columns (Qiagen). Then cRNAs prepared from the RNAs of liver tissues using protocols provided by Affymetrix were utilized to hybridize Rat Genome 230 2.0 Array (GeneChip, Affymetrix) comprised of 31,099 probe sets. In order to analyze the microarray data, we first applied the ANOVA test to filter for differentially expressed genes (*p-value < 0.001, q-value < 0.01*). 545 probe sets are identified as differentially expressed. Given the assumption that a significant set of co-expressed genes can characterize some critical transcriptional dynamics and biological significance, we explored the potential of our prior work to identify highly co-expressed gene clusters [16]. Specifically, the algorithm performs a consensus clustering and a trivial cluster removal procedure resulting in four significant clusters composed of 153, 64, 52, and 83 probesets (or 132, 42, 46, 70 genes respectively–some probesets are unmapped) that characterize four critical circadian dynamics of the homeostatic system in rat liver (Figure 1). The gene list and expression levels for each probe set are provided in the Additional file 1. Additionally, in order to test whether our identified genes express in a circadian manner, we applied the Fisher’s exact g-test which is discussed extensively in Wicher et al. [21] to assess the significance of the periodic transcription patterns. From the original expression vector of each probeset with 3 replicates at each time-point, a new vector is created for the test where replicates at time-point t (hr) are randomly distributed to three consecutive time-points t, t + 24, and t + 48. The result is that with q-value <0.01, all 352 selected probesets in 4 clusters pass the test, implying that these 290 genes do reflect periodic expression patterns. Thus they are so-called as circadian-relevant genes in this context.

The detailed dynamics of the four transcription patterns are shown in Figure 1 with the average expression profiles of all probesets in each corresponding cluster. Functional annotation was performed to develop a succinct description of the key roles of each pattern. In brief, pattern 1 (132 genes) is composed of genes whose activity peaks in the morning (ZT0 ~ 9:00 am) and is enriched by functions associated with metabolism, including (i) amino acid metabolism such as nitrogen metabolism (*car14, car5a, cth, gls2*), alanine, aspartate and glutamate metabolism (*abat, agxt2, gls2, loc641316*), glycine, serine and threonine metabolism (*agxt2, cth, gldc*), arginine and proline metabolism (*agmat,gls2*), beta-alanine metabolism (*abat, dpys*), and (ii) carbohydrate metabolism such as glycosaminoglycan degradation (*gns, gusb*), propanoate metabolism (*abat,acacb*), starch and sucrose metabolism (*gusb, pygl*). In addition, many other genes related to primary metabolism and are known to oscillate in a circadian manner e.g. *bmal, gcjr, dhtkd1, cth, gaba, gldc, kynu, akr1c18*[22], *chka and agxt*[23].

Pattern 2 contains 42 genes whose expression levels reach the maximum during the day time (ZT2 ~ 11:00 am) and appears to approach baseline before the dark phase. This pattern consists of genes with diverse functions including energy production (*ppargc1a, coq10b*), cell-cell junction (*tanc1, epb4.1 l5*), and membrane structure (*ubr4, tmem106b*). The genes associated with cell junction and component of membrane play a critical role in the integrity of the endothelial barrier function. Additionally, this pattern also contains several transcription factors involved in regulating circadian dynamics e.g. *CREM* (a transcription factor involved in output clock function and melatonin synthesis in the mouse [24]), KLF9 [25]. In a similar manner, 46 genes in pattern 3 display a maximum activity during the light phase and are primarily composed of genes associated with the immune system. Genes in this temporal class are involved in the Chemokine signaling pathway (*chuk, gnai3, stat3*), RIG-I-like receptor signaling pathway (*chuk, ddx3x*), and Toll-like receptor signaling pathway (*chuk, myd88*). Also, there are numerous genes that were shown to have circadian dynamics and related to immune function in previous studies such as *btg1*[26], *sfpq*[27], and *fkbp5*[22]. Finally, pattern 4 (70 genes) exhibits a reduced activity during the day time and reaches its peak activity around midnight. It is characterized by functions associated with DNA replication and repair, such as mismatch repair (*rfc2, rfc5*), DNA replication (*rfc2, rfc5*), and non-homologous end joining (*rad50*). Furthermore, *timeless*[28] and *calm1*[26] are also known as genes with circadian rhythmicity that vary within this pattern.

### Putative transcriptional regulators controlling circadian dynamics

In order to gain insights into the underlying regulatory mechanisms of circadian rhythms, we first analyzed the promoter regions of these putatively circadian-regulated genes in an attempt to predict putative transcriptional regulators and potential regulatory relationships. From 290 genes (of 352 probesets) in four transcription patterns, we further analyzed 134 genes that have sufficient information of orthologous promoters (at least 3 orthologous promoters) (see Methods). Table 1 shows identified putative transcriptional regulators associated with each circadian pattern. Since transcription factors are characterized by pleiotropic effects, it is reasonable to observe an overlap across the regulation of various transcription patterns [29]. While comparing these regulatory combinations, we observe that some TF families seem to be common regulators for all rhythms, suggesting that circadian dynamics of these genes may be regulated by a common underlying regulatory mechanism. These TFs are shown in bold in Table 1. Since they are commonly present as transcriptional regulators across all four patterns, they may be involved in the control of dynamics of all circadian-relevant genes identified in the homeostatic system. On the other hand, clock TFs are hypothesized to be transcriptional regulators relevant to the regulation of circadian dynamics, however, because these common TFs may or may not be clock TFs, it is possible that genes encoding them are regulated by clock TFs and in turn they regulate their target genes to express in a circadian manner. This proposed assumption is further tested in following sections.

**Table 1:** Putative transcriptional regulators controlling circadian-relevant genes

**Circadian patterns**	**Putative transcriptional regulators**
1	63 genes / 132 genes^*^
	AP1R, AP4R, CAAT, CLOX, **CREB**, **E2FF**, **ETSF**, **EVI1**, FKHD, GATA, GREF, **HOXF**, **MYBL**, MYT1, **NKXH**, **NR2F**, OCT1, PLAG, RUSH, **RXRF**, **SORY**, STAT, XBBF
2	15 genes / 42 genes
	AHRR, CLOX, **CREB**, CTCF, **E2FF**, EREF, **ETSF**, **EVI1**, FKHD, GCMF, GLIF, HAND, HEAT, HOMF, **HOXF**, **MYBL**, **NKXH**, **NR2F**, OCT1, PARF, PAX5, PERO, **RXRF**, **SORY**, SP1F, XBBF
3	24 genes / 46 genes
	AP1R, **CREB**, **E2FF**, EGRF, **ETSF**, GATA, HEAT, **HOXF**, IRFF, **MYBL**, **NKXH**, **NR2F**, PERO, **RXRF**, STAT, TBPF
4	32 genes / 70 genes
	**CREB**, **E2FF**, **ETSF**, **EVI1**, **NR2F**, **RXRF**, **SORY**, SP1F

^*^:number of genes with orthologous promoter information vs. the total number of genes in the group; TFs shown in bold are those present as regulators in at least three over four groups of genes.

### Transcriptional patterns of clock-controlled TFs and their temporal organization

**Figure 2:**
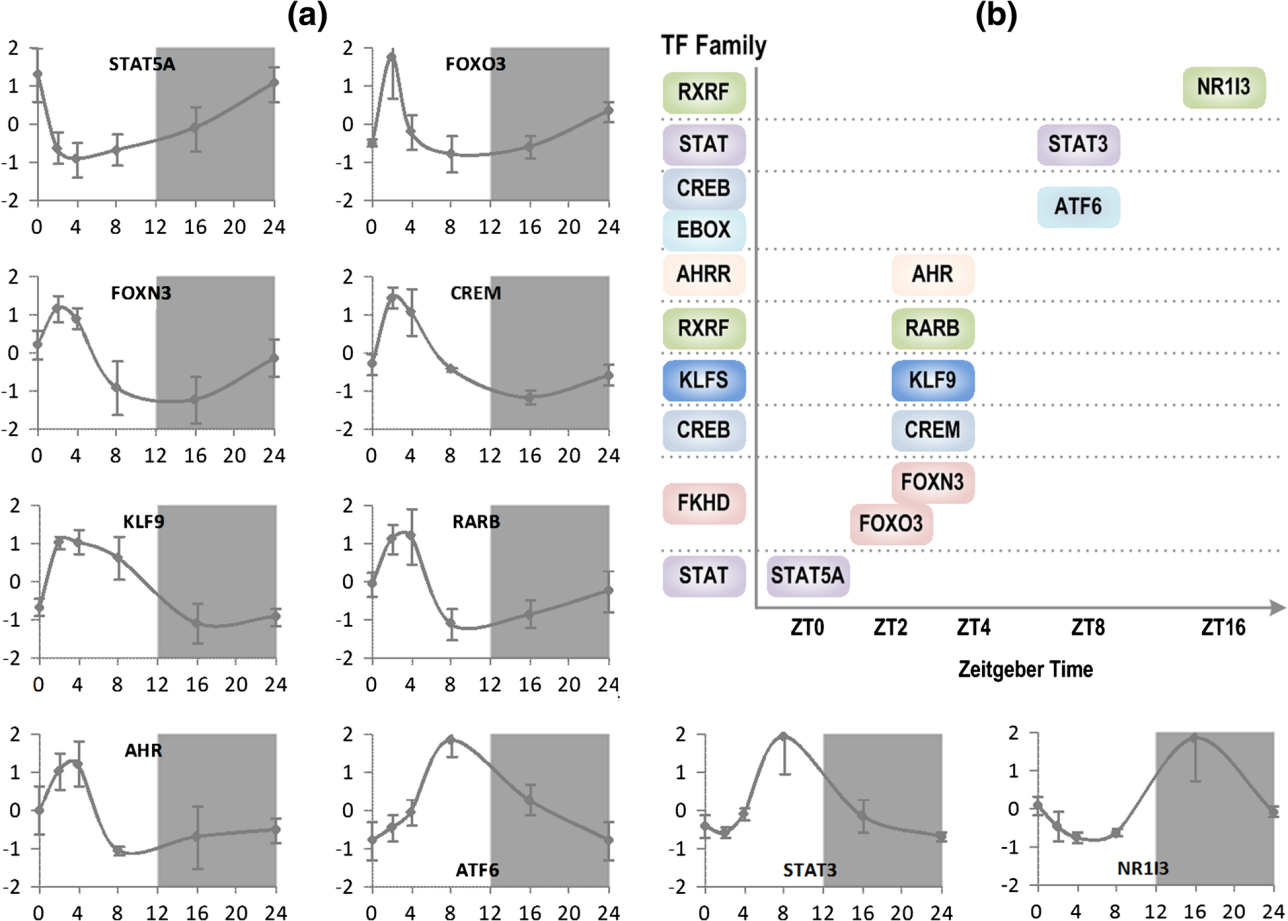
**Transcription patterns of additional clock-controlled TFs. (a)** Expression profiles of each TF in the homeostatic rat liver; the horizontal axis is the zeitgeber time (hr) and the vertical axis is the z-score normalized expression levels. Error bars are two standard deviations of expression values at each particular time-point. **(b)** The temporal organization of clock-controlled TFs. TFs with similar binding sites are combined into a TF family in the computational context. They are arranged following their corresponding peak circadian times.

To further investigate the underlying regulatory mechanisms of circadian rhythms, we explored the hypothesis that CLOCK:BMAL1, a core clock transcription factor family including CLOCK, BMAL1, NPAS2 [30], is responsible for the regulation of circadian rhythms through *cis*-regulatory modules by coordinating with other TFs whose dynamics are clock controlled. By assuming that TFs which are target genes of CLOCK:BMAL1 and also differentially expressed in homeostatic rat liver are potentially clock-controlled TFs (ccTFs), we scanned the list of 545 differentially expressed probesets to identify additional clock-controlled TFs (see Methods). A list of 10 additional ccTFs (distributed in seven TF families including STAT, FKHD, CREB, RXRF, KLFS, AHRR and EBOX) was identified. These transcription factors have dynamics that, when plotted in circadian zeitgeber time (ZT) where ZT0 (~9:00 am) is defined as the onset of diurnal activity, show an interesting progression of their peak activities. The plot of clock-controlled TFs vs. their peak circadian time and the individual profiles of the ccTFs are shown in Figure 2. Worth noting is the fact that many of these ccTFs exhibit similar transcriptional patterns. Although there appears to have no relationship between transcriptional levels and TF activity, this similarity may propose some cooperation in the transcriptional regulation of circadian-relevant genes.

### Putative clock TFs regulating the transcription of circadian-relevant genes

As it has been hypothesized that TFs usually regulate gene expression in a combinatorial manner rather than in isolation [31,32], clock TFs, including the core clock TFs and clock-controlled TFs, are also hypothesized to cooperate to regulate the transcriptional levels of circadian-relevant genes. We thus searched for significant groups of clock TFs that are significantly over-represented on the corresponding promoter set of genes in each pattern. From 290 selected genes in four circadian patterns, 234 genes have promoter annotation in Genomatix database (see Supplementary for the gene list). 620 Rattus norvegicus's promoter sequences were extracted. For each circadian pattern, a list of common sets of clock TFs were identified and only those that are statistical significant compared to the GC-matched background are selected (see Methods) (Table 2). A union of these TF sets would provide a putative set of clock TFs associated with the regulation of corresponding genes characteristic of each circadian pattern. Additionally, we tested whether the regulation of the common set of transcriptional regulators identified using the foot-printing techniques in Table 1 is influenced by clock TFs (indicated in boldin Table 1). Results show that two sets of clock TFs including KLFS_STAT (*p-value < 0.037*) and FKHD_KLFS (*p-value < 0.041*) are likely to be relevant to the regulation of the set of common regulators L. This implies that genes in circadian pattern 4 may be indirectly regulated by clock TFs.

**Table 2:** Putative clock TFs involved in the transcriptional regulation of circadian-relevant genes

**Circadian patterns**	**Sets of regulators**	**p-value | q-value**	**Sets of regulators**	**p-value | q-value**
1 109/132 genes^*^	CREB, RORA, STAT	0.0078 | 0.0616	FKHD, PARF, RXRF	0.0377 | 0.0877
	CREB, RORA, RXRF	0.0116 | 0.0616	RORA, RXRF, STAT	0.0409 | 0.0877
	PARF, RORA	0.0191 | 0.0753	CREB, FKHD, RORA	0.0473 | 0.0877
2 27/42 genes	AHRR, CREB, STAT	0.0077 | 0.0616	EBOX, CLOCK:BMAL1	0.0303 | 0.0877
	AHRR, CREB, EBOX	0.0079 | 0.0616	AHRR, RXRF, STAT	0.0327 | 0.0877
	AHRR, CREB, KLFS, RXRF	0.0130 | 0.0616	AHRR, KLFS, STAT	0.0482 | 0.0877
3 39/46 genes	CREB, EBOX, RXRF, STAT	0.0428 | 0.0877		
4 59/70 genes	None			

^*^:number of genes with promoter information vs. the total number of genes in the group.

## Discussion

### Identified circadian dynamics and biological significance

The liver expresses a diverse set of genes and previous research indicated that many hepatic genes are under direct or indirect circadian control [22]. Despite the fact that unsupervised pattern identification methods were used to identify dominant co-expressed gene sets, our results show that selected genes oscillate in a circadian manner, as shown in Figure 1. Functional characterization reveals that enriched functions are associated with metabolism, energy production, immune system and DNA replication and repair.

The liver plays an important role in the orchestration of metabolic functions such as the regulation of metabolic fuels, protein synthesis, iron and vitamin storage, as well as nutrient intake [33]. As might be expected, animals subjected to a light–dark schedule display pronounced rhythms in glycogen content, with a peak occurring late in the night, following the main period of food intake. This is also in agreement with genes identified in pattern 1, where enriched metabolic functions, especially glucose metabolism, reach their peak during the dark phase. Since the dark phase represents the active period for rats, where they are moving and eating, this increase in metabolism is indicative of both energy burn to fuel muscle usage, as well as digestive effects on the liver, and an influx of glucose and other amino acids to be processed through glycogen production and the urea cycle. Because the current study does not differentiate from fed animals and fasted animals, it is impossible to determine how much increased metabolic activity is due to demand (movement), versus supply (digestion). In addition, it has been suggested that the expression patterns of circadian rhythms in peripheral organs such as the liver are the result of delayed feedback loops of gene products from circadian regulators, suggesting that upstream regulation in addition to direct transcription factor binding can occur up to 6 hours prior to the peak of gene expression [34].

Energy production is also time-of-day dependent. It is reported that the production of ATP in rat liver is also regulated by the circadian rhythm and is lower in the dark phase [35]. Membrane integrity and cell to cell junctions are another critical circadian function that has been previously hypothesized to be important during rest [36], and contain significant circadian regulation [25]. Circadian pattern 2 is made up of a diverse array of genes whose functions are related to energy production and membrane structure, but are up regulated during the day. Many of these metabolic genes are associated with mitochondrial biogenesis specifically, which is a process that has been previously shown to occur during the resting phase of the circadian cycle [37]. As rats are nocturnal, their day cycle is indicative of their sleep cycle, this is in prior agreement and it suggests that mitochondrial biogenesis is a major liver function during the rest portion of their circadian rhythm. Genes that are associated with apoptosis are also found within this pattern such as*bclaf1*, *rarb*, *and dapk1* and are further indication of cellular repair–cells which are too damaged to be simply repaired are killed and replaced in order to prepare the host for another day of activity.

Immune system functions, including lymphocyte proliferation, natural killer cell activity, humoral immune response, absolute and relative numbers of circulating white blood cells and their subsets, cytokine levels, and serum cortisol, are known to be entrained by circadian cues [27]. Also, it has been previously reported that TNFα secretion was significantly increased in burn vs. sham isolated splenocytes cells only when injury took place in the morning [38] further verifying that the response to injury is time-of-day dependent as a result of the circadian variability of the immune functions. This is confirmed by the selected gene set exhibiting pattern 3 including*btg1*, *sfpq*, *fkbp5*, which is primarily related to immune function, and has consistent dynamics with previous works [22,26,27]. However, further analysis of the dynamics observes a peak in immune function at the end of the day (ZT8 ~ 5:00 pm), which is the time rats are beginning to awaken. Over the course of the night, when the rats are active, immune function gradually decreases, until it reaches a minimum when the night ends, and the rats rest. This implies that there may be a recovery mechanism associated with sleep that allows for the rejuvenation of immune function in order to ward off infection during active periods.

The strategy of adaptive circadian clocks could be also timing of UV-sensitive cellular processes to occur at night to avoid UV-induced damage [39]. Our analysis showed that functions related to DNA replication/repair (pattern 4) achieve a maximum activity around midnight, which is consistent with previous works [28]. However, unlike the previous patterns, the circadian dynamics of these genes are not dependant on the habits of the host animal, but rather upon external conditions related to the environment. Unlike the other patterns, the genes contained in pattern 4 do not map to specific transcription factors directly, which suggests that this group is under a different form of control than the others. The expression pattern of pattern 4 is similar to the late *cry1* phase of the circadian rhythm [40], which were identified to be under the control of newly identified D boxes that overlap with E-boxes to provide delayed expression of genes which contributes to rhythmicity. Furthermore, the *cry1* expression pattern, whose phase resembles cluster 4, is heavily dependent on other circadian regulators for its robustness [34], which suggests that pattern 4 may be controlled through upstream transcription factor regulators through their gene products, in addition to direct TF binding.

### Putative regulatory motifs of circadian-relevant genes

**Figure 3:**
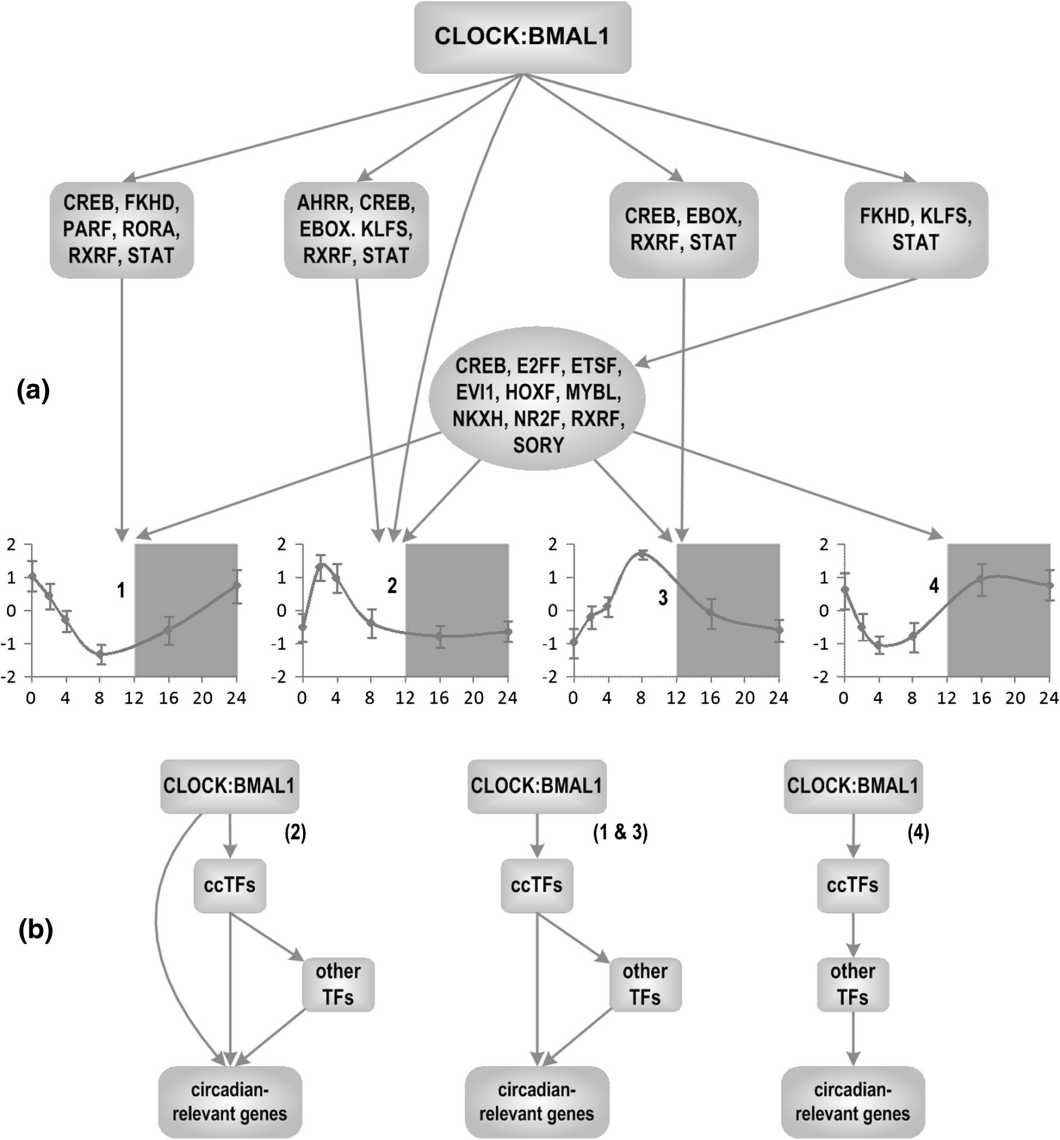
**Putative regulatory program of circadian-relevant genes (a) and proposed regulatory motifs (b) ccTFs are clock-controlled TFs.** Regulation of rhythm 2 is corresponding with the first regulatory motif rhythm 1&3 with the second and rhythm4 with the last one.

In order to investigate the underlying regulation behind the co-expressed circadian genes, promoter analysis was applied to identify key transcriptional regulators associated with the regulation of each pattern. The initial assumption for this analysis is that core-clocks TFs are responsible for the regulation of circadian rhythms through coordination with other TFs whose dynamics are clock controlled (ccTFs). However, besides clock TFs (including core-clock TFs and ccTFs) there could be other TFs also involved in the regulation of transcriptional patterns of these circadian-relevant genes. As such, we first explored the underlying hypothesis in comparative genomics to predict relevant transcriptional regulators using the foot-printing technique (Table 1) [41,42]. Subsequently, we explored the initial assumption above by searching for statistical significant sets of clock TFs that are over-represented on the corresponding promoter set of genes in each pattern (see Methods). PARF and RORA are also included in this search because they are widely known to be regulated by core-clock TFs (Table 2–the 3 left rectangles in Figure 3a). Furthermore, to exhaustively examine if any set of clock TFs relevant to the regulation of common regulators of circadian genes, we applied the latter methodology to the set of common regulators identified in Table 1 (TFs in the circle of Figure 3a). Results showed that a set of three clock-controlled TFs (STAT, FKHD, and KLFS) are associated with their regulation. These results are integrated and displayed in Figure 3a, which consists of TFs that are regulated by CLOCK:BMAL1 and directly regulate individual circadian patterns, as well as those that are regulated by ccTFs and affect the dynamics of all four patterns.

Simplified networks are shown in Figure 3b, which simply condense the transcription factors into three categories: core clock transcription factors (CLOCK:BMAL1), clock controlled transcription factors (ccTFs) and other transcription factors. Genes which exhibit transcription pattern 2 show the most circadian influence, with the corresponding promoter set containing enriched binding sites for the core clock TFs, ccTFs, and other TFs. Meanwhile genes following pattern 4 has the least circadian influence, being only controlled indirectly through two levels of regulation by the core clock transcription factors, likely through their gene products [40]. Because of the overlapping levels of regulation associated with pattern 2, it can be expected that disruptions to the circadian cycle will be less effective at disrupting its gene expression, as those layers of regulation could potentially provide redundancy that preserves circadian gene expression. Meanwhile, pattern 4, can be putatively said to be more sensitive to circadian disruptions, due to the lack of backup regulation. This is supported by the fact that pattern 4 has a similar expression to the *cry1* gene, which is a core circadian regulator known for its sensitivity to changes in the overall circadian rhythm. Further, since pattern 2 is representative of host repair during rest, it makes sense that it would be protected against disruption: proper sleep is critical to health and disease recovery [43], and the redundancy of the host repair genes may reflect that need. Similarly, pattern 4 is associated with DNA repair, which has been shown to be severely affected following injury due to the increased presence of reactive oxygen species [44,45], known to damage DNA. Thus the putative sensitivity of this pattern to circadian disruption may be a result of altered DNA repair patterns during periods of stress. Genes in pattern 1 and 3 are both under similar circadian regulation, despite being significantly separated in circadian time, as discussed previously. These patterns primarily relate to metabolism and innate immunity, two functions which have been shown to be closely linked through a phenomenon known as hypermetabolism [46]. These functions show significant circadian sensitivity, and because of their peaks at different circadian times (ZT0 and ZT8), it is possible that due to different innate immune and metabolic states over the course of the circadian day, the time of injury can significantly impact the severity of the immune response and the onset of hypermetabolism. The hypothesis that circadian rhythms strongly impact immune responses following injury has been postulated before [47], but further experiments are needed in order to conclusively link the functions, and dynamics of genes in pattern 1 & 3 to injury responsive elements.

### Dynamic and functional agreement between clock-controlled TFs and their associated gene sets

In Figure 2, it is worth noting that the transcription factors that have early circadian time peaks (ZT0 to ZT4), namely STAT5A, FOXO3, FOXN3, CREM, and RARB are all associated with the regulation of genes in transcription pattern 1, whose genes also peak very early in circadian time. Transcription factors which peak at somewhat later times (ZT4-ZT8) include RARB, KLF9, AHR, ATF6, and STAT3, which have all been associated with pattern 2. This pattern peaks at a later time period than the first pattern, and this is reflected in the shift in circadian time peaks in its transcriptional regulators. Finally, the transcription factors which peak at the latest circadian times (ZT8-Z16) include ATF6, STAT3, and NR1I3, which are all associated with pattern 3. This pattern peaks at the latest time point following the diurnal onset, and again, this is reflected in the shifted circadian time peaks of its regulators. In general, many of transcription factors control the same set of genes exhibiting a circadian pattern express a very similar circadian profile.

Though many transcription factors have been identified, the three clock-controlled TF families which control the functions of all of the patterns through binding sites on non-clock controlled TFs are FKHD, KLFS, and STAT (Figure 3). The FKHD transcription factor family includes the Fox-O transcription factor, which is well known for its protective role against oxidative stress within the body [48]. Similarly, KLFS is well known for its role in the regulation of anti polymicrobial bacteria activity within the body and metabolism in liver [25,49], while STAT has already been identified as a critical transcription factor during the outcome of sepsis [50]. In particular, KLF has been strongly associated with liver metabolism in particular [25], which is reinforced by its regulation of cluster 2, which has many metabolic genes associated with it. Because KLF is strongly associated with CLOCK and BMAL, its presence as a regulator of these oscillatory gene clusters suggests significant circadian regulation of these functions. Together these three mediators and their functions indicate that the overarching control of clock TFs to the patterns indirectly through non clock-controlled TFs is controlled primarily by the innate immune response, and may serve to coordinate the metabolic functions and toll like receptor functions found in the patterns in the event of an innate immune response.

A TF family that is considered as a common regulator of genes across all four circadian patterns is CREB. It has been previously recognized to be prototypical stimulus induced in function [51], and therefore plays a wide variety of roles in different tissues. However, it has been strongly linked to functional circadian regulation throughout the body, and thus it is not surprising that CREB regulatory elements are found in all four patterns, both directly and indirectly regulated by CLOCK:BMAL1. Unique clock controlled TF families that regulate genes in pattern 1 are PARF and RORA. PARF includes well known clock TFs (e.g. DPB, TEF) which are directly targets of CLOCK:BMAL1 [52,53]. RORA is another well known circadian transcription factor family, which has also been shown to play a role in metabolism in many tissues, including the muscle and liver systems [54,55]. Additionally, RXRF transcription factors represent a family of nuclear receptors that target a variety of signaling pathways, which are primarily related to metabolism, cell differentiation, and cell death [56]. Thus, much like others associated with pattern 1, RXRF factor family has significantly overlapping functions in the literature to the genes that they control. Unique transcription factor families that regulate genes in patterns 2 and 3 include AHRR and EBOX. AHRR, which is a unique clock transcription factor family associated with pattern 2, has been associated with the regulation of cell adhesion and matrix formation [57], which is in agreement with the functions of genes in pattern 2. EBOX, which is associated with the regulation of genes in both patterns, has implicit functions in innate immunity, and it has been shown that the mechanism by which TNF-α, a well known mediator of the inflammatory response, suppresses clock genes is through interference with EBOX mediated transcription [58]. EBOX transcription factors have also been shown to be key regulators of the circadian rhythm, including well known circadian genes and clock controlled transcription factors [59]. Thus, both EBOX and AHRR not only have functions that are similar to those of their respective gene sets, but also have prior association with circadian regulation, which suggests that many of the regulators which control innate immunity and metabolism within the liver are at least partially under the regulation of the circadian rhythm.

### Alternative factors relevant to the regulation of circadian genes

Our promoter analysis of transcription factors is based on the assumption that clock TFs are mainly responsible for the transcriptional dynamics that are observed over 24 hours within the liver. However, other factors, including SCN stimuli and food intake in the form of releasing glucocorticoids from the adrenal gland, light stimuli, and heat, have been known to impact circadian rhythms [60]. This is particularly important in the context of genes in pattern 4, whose functions are closely related to DNA repair and maintenance and which peaks during the night, thereby repairing any UV damage that occurs.

Among transcription factor families identified from Table 1, GREF represents a known transcription factor responsive element that is stimulated by glucocorticoids [61]. Glucocorticoids are well known molecules that are coordinated by the SCN in order to entrain peripheral tissues in a circadian manner [62]. Since genes in pattern 1 are controlled by this transcription factor family, it suggests that many liver metabolic functions may also be regulated by the SCN. This fits the current paradigm of SCN regulation, since the SCN has been associated with the regulation of feeding patterns [63]. Therefore, by controlling metabolic enzymes within the liver to be most active at ZT0, the SCN can coordinate metabolic functions to coincide with feeding patterns.

Another alternative regulatory mechanism for circadian rhythms is through light. Although there is no direct light input to liver, light may trigger a signaling cascade which eventually activates some functional TFs in liver. This activation requires the aid of protein kinases (PKA) [64], which activate CREB and allow it to translocate to the nucleus for transcriptional regulation [65]. This two-step activation controls all four circadian pattern motifs through CREB factors, and may, in addition to CLOCK:BMAL1, be responsible for the diurnal activity observed within the liver. The importance of light in the sleep/wake cycle has been well established [66], and thus it is not surprising that the circadian activity of the liver may be entrained to the same light/dark rhythms that govern sleeping cycles in mammals.

**Figure 4:**
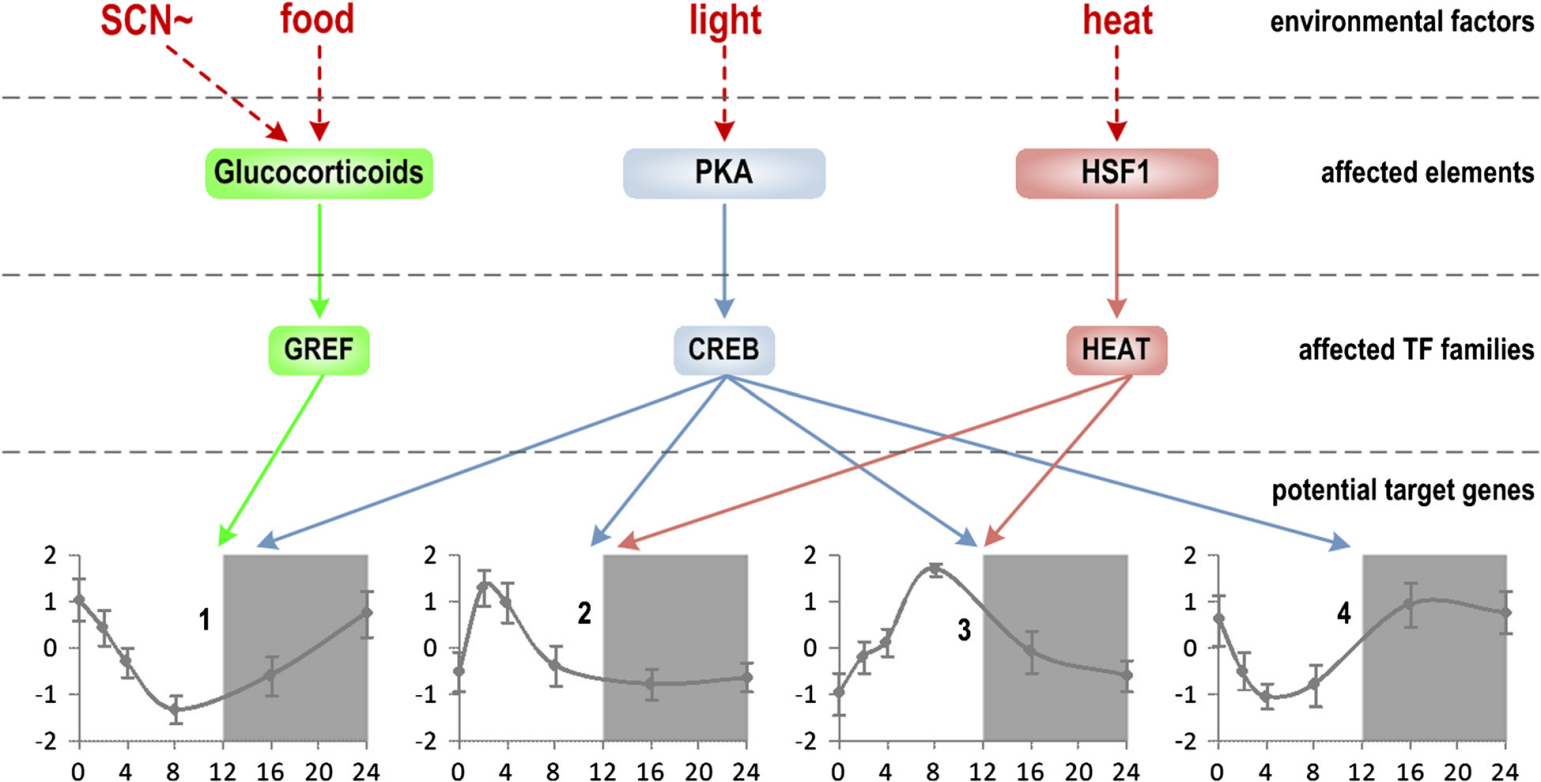
**Predictive effects of alternative factors relevant to the transcriptional regulation of circadian-relevant genes.** ‘Affected elements’ are factors affected by environmental factors proposed in literature. ‘Affected TF families’ are those provide the link between the effects of external environmental factors and their potential target genes proposed by our computational framework.

Finally, genes in pattern 2 & 3 are also regulated by the HEAT transcription factor family, which is known to be responsive to temperature cues [67]. These cues have been shown to regulate the circadian rhythm, and thus, it is unsurprising that the innate immune response and cell repair mechanisms are tied to heat sensitivity. The link between heat shock transcription factors on a circadian level and innate immune response genes may be a part of why burn injuries often illicit severe immune responses in the rat liver [68]. Overall, these three alternative stimuli likely act in parallel with clock transcription factors, in order to give the circadian rhythm plasticity in its response to various environmental challenges. Figure 4 reinforces the fact that environmental factors do affect the circadian transcriptional regulation and provides a link to reveal how they affect as well as which genes are potential targets of which environmental factors. Further work should focus on the degrees of control that each of these stimuli has on the overall response of the circadian pattern, using a systems biology approach to elucidate and refine circadian networks and the ways in which they crosstalk.

## Conclusions

Microarrays were used to characterize the gene expression within rat livers over 24 hours, and various computational tools have been applied to identify and distribute differentially expressed genes from the microarray data into four distinct patterns. Pattern 1 is primarily related to central metabolism with the peak at circadian time ZT0. Pattern 2 is related to cell repair and cell junction formations, and peaks at circadian time ZT4. Pattern 3 is primarily related to the innate immune response and peaks at circadian time ZT8, while Pattern 4 is related to DNA repair and peaks at circadian time ZT16. The study also proposed a unique regulatory structure for circadian rhythms in the liver, which aims to predict the ways in which circadian patterns can potentially impact injury. By using techniques from promoter analysis coupled with the proposed hypothesis of clock TF regulation, a set of transcription factors and putative regulatory structure have been established which show to be consistent in agreement with functional and temporal annotations. These transcription factor dynamics appear to play a strong role in circadian regulation, and therefore potentially impact the liver’s response to environmental challenges at different circadian times. In conclusion, our study aims at (1) unsupervised identification of circadian-relevant genes and (2) exploration of potential regulatory mechanisms relevant to the transcriptional regulation of circadian genes. While doing that, we eventually proposed a promising computational framework that can predict relevant transcriptional factors and putative regulatory structure of circadian genes in a genome-wide manner, establishing a platform towards large-scale comparisons of the regulation in homeostatic systems across multiple tissues and/or conditions.

## Methods

### Animal model

Male Sprague–Dawley rats (Charles River Labs, Wilmington, MA) weighing between 150 and 200 g were utilized for this study. The animals were housed in a temperature-controlled environment (25°C) with a 12-hour light–dark cycle and provided water and standard chow ad libitum. Because the animals are nocturnal, this establishes a 12 hour LD cycle for the animals, where ZT0-ZT12 represents the resting portion of the day, while ZT12-ZT0 represents the active portion of the day. All experimental procedures were carried out in accordance with National Research Council guidelines and approved by the Rutgers University Animal Care and Facilities Committee.

Animals are sacrificed at 9 am, 11 am, 1 pm, 5 pm, 1 am, and 9 am (~0, 2, 4, 8, 16, and 24 hr zeitgeber time). Animals were originally used as part of the study by Yang et al. [68] to analyze gene expression following burn injury, which dictates the choice of time points. Ideally, future works would aim at sampling the latter parts of the night and day phases equally to observe trends throughout the time course. Liver tissues were collected and frozen for microarray analysis (n = 3 per time point per group). The tissues were lysed and homogenized using Trizol, and the RNAs were further purified and treated with DNase using RNeasy columns (Qiagen). Then cRNAs prepared from the RNAs of liver tissues using protocols provided by Affymetrix were utilized to hybridize Rat Genome 230 2.0 Array (GeneChip, Affymetrix) comprised of 31,099 probe sets.

### Microarray data analysis

Genome expression data analysis included normalization, filtering for differential expression, and clustering. DNA chip analyzer (dChip) software [69] was used with invariant-set normalization and perfect match (PM) model to generate expression values. The analysis of variance (ANOVA) test implemented in R [70] was applied to filter differentially expressed probesets in the dataset with *p-value* < 0.001. At this step, to control the false discovery rate the method proposed by Storey [71] was used with a positive false discovery rate *q-value* cutoff of 0.01. Due to a limitation of data, such strict thresholds were used and thus several well-known circadian genes were not present in the filtered gene set (see Additional file 1, sheet ‘filtering’). Subsequently, consensus clustering [16] was applied in order to identify, in an unsupervised manner, coherent clusters of co-expressed genes with *p-value < 0.001*. The biological relevance of the intrinsic responses was also characterized by evaluating the enrichment of the corresponding subsets in circadian rhythm specific pathways using KEGG database through ARRAYTRACK (*p-value < 0.05*) [72] and characterizing functionally individual genes.

### Promoter extraction and processing

Promoters of genes including all transcript-relevant alternative promoters were extracted from a rich database of promoter information. If there is no experimentally defined length suggested by Genomatix, a default length, 500 bp upstream and 100 bp downstream of the transcription start site which is the most enriched region of transcription factor binding sites (TFBSs) according to Genomatix, is used [73]. In order to accelerate the process of identifying putative transcriptional regulators, promoters are pre-processed as in [74]. Specifically, MatInspector [75] is applied to scan for PWM matches on those promoter sequences using optimal parameters from MatBase which ensures that the minimum number of matches found in non-regulatory sequences i.e. the false positive matches is minimized [73]. Each promoter of a gene is then re-modelled to become a list of TFBSs ordered by their local positions on the promoter sequences and represented by corresponding TF names along with their binding orientations. The conversion supports for fast search the presence of a TFBS or a set of TFBSs on promoter sequences [74].

### Prediction of relevant transcriptional regulators

In order to predict putative TFs relevant to the transcriptional regulation of circadian-relevant gene sets, we explored the basic underlying assumption of comparative genomics which states that functional regions evolve in a constrained fashion and therefore at a lower rate than non-functional regions [41,42]. Consequently, orthologous promoters of each gene are further extracted. Each promoter P is now characterized by a set of orthologous promoters from the same gene of other vertebrate species, if available (e.g. *Homo sapiens, Macacamulatta, Pantroglodytes, Musmusculus, Equuscaballus, Canis lupus familiaris, and Bos Taurus*). To be consistent in the search for conserved regions on promoter sequences in order to identify putative TFBSs, we eliminate those that do not consist of more than two orthologous promoters [76]. DiAlign TF [77] with default parameters (core similarity: 0.75, matrix similarity: optimal threshold for each PWM suggested from MatBase) was applied to identify conserved regions on promoter P. We next apply MatInspector [75] to scan for all physical TFBSs and only keep those that locate on conserved regions. Subsequently, given a set of genes, TFBSs that are enriched with a common threshold (70% in this study) are identified and considered as TFs relevant to the transcriptional regulation of the gene set.

### Identification of clock-controlled transcription factors

Besides TF families composing of clock TFs identified from literature (CLOCK:BMAL1, PARF and RORA [78]), we hypothesized that transcription factors that contain CLOCK:BMAL1 binding sites on their promoters and also differentially expressed in the homeostatic system are likely to be clock-controlled TFs. After identification of differentially expressed genes from the entire dataset, MatInspector [75] was applied to scan for TFBSs present on corresponding promoters of each genes. Genes that contain CLOCK:BMAL1 binding sites and are also reported as transcription factors in MatBase [73] are considered as clock-controlled TFs.

### Identification of common sets of clock transcription factors

It has been noticed that TFs in higher eukaryotes regulate gene expression in a combinatorial manner rather than in isolation [31,32]. Consequently, to explore the underlying mechanisms of clock TFs in the relation to the regulation of these circadian rhythms, we hypothesize that significant sets of clock TFs present on promoter sets of co-expressed circadian-relevant genes are more likely to be coordinated regulators in modulating the transcriptional process of circadian rhythms. We thus applied the breadth first search technique to identify set of clock TFs that are commonly present on the promoter set of genes in each circadian pattern. Since a gene may have multiple alternative promoters, if a set of clock TFs present on any promoter of the gene it is considered as presence on that gene. The procedure first identifies all potential clock TFBSs that are commonly present on the corresponding promoter set and then search for all possible combinations of those TFBSs (common level of 70%). Statistical significance of each common set of clock TFs is evaluated to select significant sets for further examination (*p-value < 0.05, q-value < 0.10*) (detailed in Additional file 1).

### Statistical significance of sets of clock TFs

In order to construct the relations of clock TFs to the regulation of circadian rhythms, we estimate the significance of common sets of clock TFs identified from the corresponding gene set of each transcription pattern vs. the background set. The procedure proposed by Bozek et al. [79] was followed to extract a GC-matched background gene set (~10,000 genes) in order to compensate the relatively high GC-content promoters of circadian relevant genes (detailed in Additional file 1).

Given a gene set G, the significance of set of clock TFs A is calculated based on a hyper-geometric *p-value* defined as follows:

$$p-\mathrm{value}\;\left(A\right)=\left(\begin{array}{l} b\\ n \end{array}\right)\;\left(\begin{array}{l} B-b\\ N-n \end{array}\right)\;/\;\left(\begin{array}{l} B\\ N \end{array}\right)$$

where B and b is the number of genes and the number of hits respectively in the background set; N and n is the number of genes and hits in gene set G, respectively. A ‘hit’ is the presence of set A on a promoter of a gene.

Additionally, to restrain the multiple testing problem in searching for significant sets of clock TFs, the bootstrap method proposed by Storey [71] was utilized to estimate the positive false discovery rates (q-value). A rate of 10% was used in this case.

## Competing interests

The authors declare that they have no competing interests.

## Authors’ contributions

TTN developed the computational methodologies and implementation and run the studies. JSAM, QY, MAA contributed to the analysis and drafting of the manuscript. MGI and FB edited the manuscript. IPA conceived the study, guided the development and analysis and edited the manuscript. All authors are reviewed and approved the final version of this manuscript.

## Supplementary Material

Additional file 1 This file includes all the information pertaining to this paper including gene names, raw expression data, binding sites, transcription factors and all relevant statistics. Click here for file
